# Diversity, Physicochemical and Technological Characterization of Elite Cassava (*Manihot esculenta* Crantz) Cultivars of Bantè, a District of Central Benin

**DOI:** 10.1155/2015/674201

**Published:** 2015-11-29

**Authors:** Abadjayé Faouziath Sanoussi, Laura Yéyinou Loko, Hyacinthe Ahissou, Adidjath Koubourath Adjahi, Azize Orobiyi, Angelot Paterne Agré, Paulin Azokpota, Alexandre Dansi, Ambaliou Sanni

**Affiliations:** ^1^Laboratory of Biotechnology, Genetic Resources and Plant and Animal Breeding (BIORAVE), Faculty of Sciences and Technology of Dassa, Polytechnic University of Abomey, 071BP28 Cotonou, Benin; ^2^Laboratory of Protein Biochemistry and Enzymology, Faculty of Sciences and Technology (FAST), University of Abomey-Calavi (UAC), BP 526 Cotonou, Benin; ^3^Department of Nutrition and Food Technology, Faculty of Agricultural Sciences (FSA), University of Abomey-Calavi, BP 526 Cotonou, Benin; ^4^Laboratory of Biochemistry and Molecular Biology, Faculty of Sciences and Technology (FAST), University of Abomey-Calavi (UAC), BP 526 Cotonou, Benin

## Abstract

Cassava is one of the staple food crops contributing significantly to food and nutrition security in Benin. This study aimed to assess the diversity of the elite cassava cultivars of Bantè district, determine the physicochemical properties of the most preferred ones as well as the sensory attributes of their major derived products (gari and tapioca), and compare them with the farmers' and processors' perception on their technological qualities. The ethnobotanical investigation revealed existence of 40 cultivars including 9 elites that were further classified into three groups based on agronomics and technological and culinary properties. Clustered together, cultivars* Idilèrou*, Monlèkangan, and* Odohoungbo* characterized by low fiber content, high yield of gari and tapioca, and good in-ground postmaturity storage were the most preferred ones. Their physicochemical analysis revealed good rate of dry matters (39.8% to 41.13%), starch (24.47% to 25.5%) and total sugars (39.46% to 41.13%), low fiber (0.80% to 1.02%), and cyanide (50 mg/kg) contents. The sensory analysis of their gari and tapioca revealed very well appreciated (taste, color, and texture) products by the consumers. The confirmation by scientific analysis of the farmers' perception on qualities of the most preferred cultivars indicated that they have good knowledge of their materials.

## 1. Introduction

Cassava (*Manihot esculenta* Crantz) is one of the most important staple food crops grown in the tropics [1, 2]. It plays an important role in ensuring food security in most of the developing world, namely, Africa, the Asian Pacific, and South America [2, 3]. Currently, more than 750 million people of which 45% of sub-Saharan Africans, rely on cassava as their primary food source [2]. All the parts of the crop are useful either for human consumption (young leaves and roots) or for feeding animal [4]. Cassava plant is well known for its ease of cultivation, adaptability to poor soil conditions, low rainfall, high temperature, and resistance to drought [2, 4, 5]. Such characteristics are especially relevant in coping with the ongoing climate change [2]. Moreover, it constitutes source of income for several billion farmers, traders, and industries [3, 6] and it contributes significantly to the economy of most tropical countries through processing into various products [7, 8].

Cassava root has many uses. The roots are processed into flour, starch, and other end products (chips, flakes, biofuel, textile, and glue) [9]. However, the uses of the roots are constrained by some factors. They are perishable; require rapid utilization after harvest [3, 9] and content sometimes detrimental levels of cyanogenic glycoside [10]. In Africa, improperly processed cassava is a major problem associated with a number of cyanide-related health disorders, particularly among people who are already malnourished [11]. Accumulation of cyanide leads to the development of goiter and tropical ataxic neuropathy, a nerve-damaging disorder that renders a person unsteady and uncoordinated [11]. Severe cyanide poisoning, particularly during famines, is associated with outbreaks of a debilitating, irreversible paralytic disorder, and, in some cases, death [11]. According to [12], the potential for poisoning is further complicated by the fact that the cyanide content of cassava is known to vary with environmental conditions, such as drought (leading to an increase in cyanogenic potential). In parallel, it is generally known that processing techniques are employed to detoxify cassava root and reduce cyanogenic glycosides to safe levels [10] and cassava products that are not adequately processed have been linked to cyanide poisoning [13].

In West Africa, particularly in Benin, several cassava cultivars exist and they are mainly consumed after processing into gari, traditional flour, lafun, and improved flour [13]. Cultivars are thus selected and cultivated by farmers based on consumers preferences in terms of quality, which is mostly governed by the final texture (particularly the friability or mealiness) and the taste of the boiled roots [14, 15]. However, the diversity, distribution, and extent of the mostly used or preferred cassava cultivars at Bantè, its high production and processing areas in Benin, are unknown. In addition, the technological aptitude of each cultivar in processing into gari (flour from grated cassava that is subsequently fermented for 1-2 days and roasted or/and dried) and tapioca (partially gelatinized roasted cassava starch, which appears as flakes or irregularly shaped granules), the traditional largely consumed products seem to be known by producers and processors but are not yet documented to be properly exploited by scientific research. The link between physicochemical properties of fresh cassava roots and farmers' perception on its technological processing aptitude is not yet established. This study initiated to fill this gap of knowledge is aimed at the following:

- assessing diversity of elite cassava cultivars in Bantè and identifying the most preferred ones based on their technological and culinary performances,
- evaluating the physicochemical composition of the most preferred elite cassava cultivars for comparison with farmers' knowledge or perception on their technological aptitude,
- determining sensory profile of the gari and tapioca produced with these most preferred cultivars.

## 2. Material and Methods

### 2.1. Study Area and Diversity Analysis

**Figure 1:**
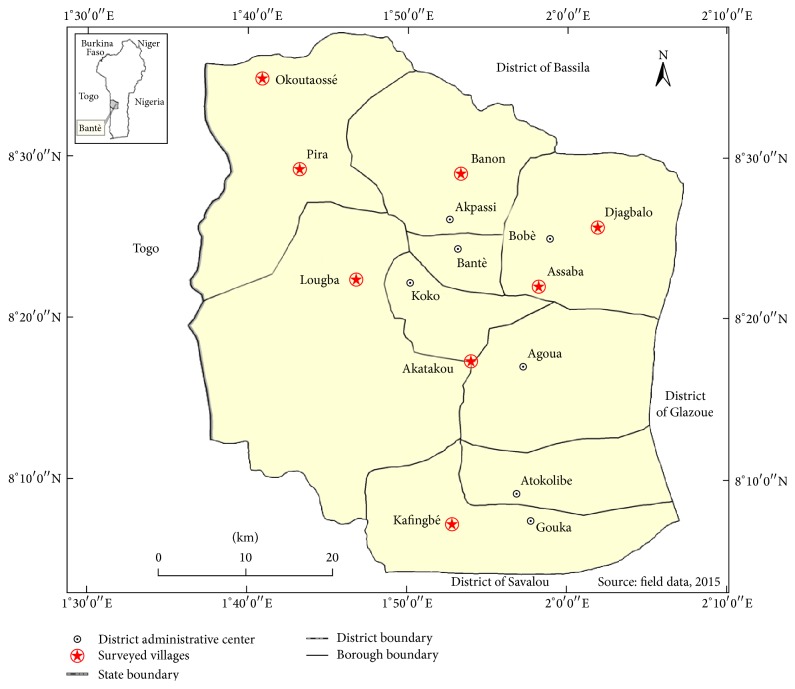
Map of the study area showing the villages surveyed.

This study was conducted in the district of Bantè located in the forest and humid region of central Benin (West Africa). It covers an area of 2695 km^2^ and is inhabited mainly by the ethnic groups Itcha [16]. The district is partitioned in 8 major villages which are Akatakou, Assaba, Bânon, Djagbalo, Kafègnigbé, Lougba, Okoutaossê, and Pira (Figure 1). For the study, these villages were selected for survey.

Data were collected during expeditions from the different sites through the application of participatory research appraisal tools and techniques, such as direct observation, group discussions, individual interviews, and field visits using a questionnaire [17, 18]. In each village, interviews were conducted with the help of a local translator. Group discussions were held with an average of 30 cassava producers (both females and males) of different ages. In each site, local farmers' associations and the chiefs of the village were involved in the study to facilitate the organization of the meetings and data collection. Cultivars used were listed and their distribution and extent were assessed using the Four Square Analysis method following [17]. The Four Square Analysis method allows classifying into four classes and in farmers' participatory way cultivars were identified in a given village base on the relative (small or large) size of the land area devoted to the variety and on the relative number (few or many) of households cultivating it [17]. Then, discussions took place on each cultivar with the view of documenting its agronomic, technological, and culinary characteristics. The Four Square Analysis method is used to identify elite cultivars (cultivated on large areas and by many households) and to assess the rate of cultivars loss.

### 2.2. Physicochemical Analysis

Cassava root samples were collected from the cassava germplasm maintained as field collection at the faculty of sciences and technology of Dassa. Collected samples were washed, the outermost “papery” layer of skin (the periderm) and the next outermost layer of each root sample, the cortex (or peel), were then removed. A metal cork-borer was used to take sample of tuber parenchyma (or flesh) adjacent to the center of the transverse section. The samples of cassava flesh were ground by Moulinex DPA1 41 and the paste obtained was homogenized and stored in a refrigerator (10°C) for later analysis of dry matter, total sugars, fibers, starches, and cyanide content.

Dry matter was determined according to AOAC method [19]. The moisture and solids content were determined by drying 5 g of flour in the oven at 105°C temperature. Every 2 hours of drying the sample is removed from oven and weighed. This was repeated until stable products weight was obtained.

Crude fiber was determined from the residue of defatted sample by keeping 5 g of sample in a muffle furnace at temperature of 900°C for 6 hours following AOAC methods [19].

Starch content was determined using the anthrone method [5, 12]. Starch extraction was carried out by adding 3 mL of 66% of perchloric acid to 0.2 g cassava mash for 20 min and then diluted into 100 mL. Two millimeters of obtained solution was placed in test tubes and mixed with 5 mL of anthrone reagent. The test tubes were placed in a boiling water bath and left for 12 min. They were then cooled and the absorbance was measured at 490 nm.

The total sugars content was determined using the colorimetric test as described by [20]. Fresh cassava samples were finely crushed and passed through filter of 0.5 mm mesh. 1 g of the crushed and filtered sample then obtained was added to 5 mL of distilled water and centrifuged. About 0.2 mL extract obtained from centrifuged sample at 2000 rpm for 10 min was added to 0.8 mL distilled water and then 0.5 mL phenol (5% (p/v)) and 2.5 mL H_2_SO_4_ extract were brutally added. After being softly homogenized the mixture was boiled at 100°C for 5 min and then cooled at darkness during 30 min. The absorbance was measured at 490 nm. The sugars content is calculated using the following formula:

(1) %Sugar=Abs−Intercept×Dilution factor×VolumeWeight of sample×Slope×10,000,

where Abs is absorbance, Dilution factor = 5, Volume = 20, Slope = 0.0055, and Intercept = 0.0044.

The method used for cyanide analysis was described by [12]. Solution of 20 g was steam-distilled in 40 mL NAOH (25 g/L) and then added to 8 mL KI (5% (p/v)). The solution was titrated with 0.02 N AgNO_3_ until apparition of light trouble.

### 2.3. Sensory Quality Determination

The sensory profile was assessed using multiple comparison test described by [21]. The panel was composed of 30 regular consumers (panelists) of gari and tapioca. Gari samples from the different cassava varieties were simultaneously presented to the panelist in plates followed by the tapioca samples. Tap water was provided for panelists to rinse mouth between samples. The samples of gari and tapioca were analyzed for various attributes such as color, taste, and texture. The sensory test was performed in naturally illuminated room. The panelists were initially trained to rank these attributes on a semistructural scale (1-2). The taste of the gari and tapioca will be as follows: 1 = sweet gari or tapioca; 2 = very sweet gari or tapioca. The color of the sample will be as follows: 1 = white color; 2 = brownish white. The texture will be as follows: the value 1 represents very dry gari or tapioca, while 2 is related to dry gari or tapioca.

### 2.4. Statistical Analysis

Cassava cultivars diversity (in terms of agronomic and technological performances) was assessed using UPGMA (Unweighted Pair-Group Method with Arithmetic Average) clustering method by considering identified cassava cultivars as individuals and evaluation parameters as variables using NTSYS-pc 2.2 (Numerical Taxonomy and Statistical Analysis) software [22] following [18].

All chemical analyses were performed in triplicate. The data was analyzed using EXCEL Spreadsheet and STATISTICA version 7.1. For physicochemical and sensory analysis parameters, descriptive statistics (means and standard deviations) were computed and *t*-test of student was performed to assess the significance (*P* < 0.05) of differences between means.

## 3. Results and Discussion

### 3.1. Diversity, Distribution, and Extent of Cassava Cultivars in Bantè

Subject to clarification of eventual synonymy, 40 cassava cultivars were identified in the 8 surveyed villages (Table 1). The number of cultivars identified per village varied from 11 to 26 with an average of 15 varieties per village. Assaba presented the greatest number of cultivars (26) whereas the fewest number of cultivars (11) was recorded in Djagbalo and Pira. Among the 40 cassava cultivars identified, some like Odohoungbo and Ôlôbêkpê were found in all villages surveyed while others like Bocconon and Bamiwômo were found in only one village.

The distribution and extent analysis revealed that, despite the existing diversity, only 9 cultivars (BEN, Idilerou, Maboussa, Monlekangan, Odohoungbo, Ôkôtiyawo, RB, Tatawili, and TMS) were found in at least one village as cultivated by many households and on large areas (Table 1). These cultivars could be considered as elite following [17]. In the absence of improved lines, elite cultivars can be used by NGOs or development projects in agricultural extension or in some diversity exchange programs between villages [9, 23].

**Figure 2:**
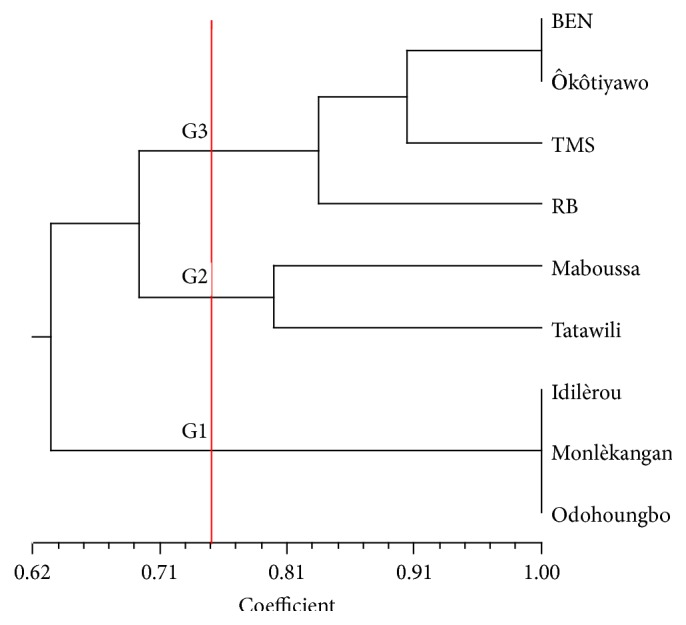
Dendrogram showing the classification of the 9 elite cultivars into three groups.

Using their agronomic performances and their technological and culinary characteristics, the dendrogram performed with NTSYS software grouped, at 75% of similarity, the 9 elite cassava cultivars identified into 3 different units G1, G2 and G3 (Figure 2). The composition of these units and their key characteristic traits are summarized in Table 2. In G1 three cultivars (Odohoungbo, Monlèkangan, and Idilèrou) cluster together with very good technological and/or culinary characteristics, high productivity, good in-ground postmaturity storage, and maturity cycle ranging from 12 to 18 months. The second group (G2) was composed of Maboussa and Tatawili cultivars. These cultivars have average productivity. G3 assembles four cultivars (RB, TMS, Okotiyawo, and BEN) characterized by high productivity, early maturity (6 to 8 months), sweet taste, and poor in-ground postmaturity storage aptitude (root rot or transformation into fiber). Farmers and processors reported that within a set of elites cultivars characterized by low fiber content, high yield of gari, and good quality of end products (gari, tapioca, and dough), high productivity and good in-ground postmaturity storage aptitude are their two key preference criteria. Processors generally buy cassava when it is still immature, growing in the field. When the plants reach maturity, they gradually harvest and process into end products depending on the market demand. In such conditions, it is not surprising that only G1 cultivars are the most preferred cultivars as reported the farmers.

### 3.2. Physicochemical Characteristics of the Preferred Elite Cultivars

Farmers and processors perceptions on some physicochemical properties of fresh cassava root of these three most preferred cultivars are reported in Table 3. Low cyanide content cassava was scored as the most bitter [15]. All the respondents recognized the super-elite cassava cultivars as having bitter taste. They could be considered as low cyanide content cultivars as highlighted by [15] for some bitter taste cassava cultivars. For almost all the respondents (82.93% to 100%), Odohoungbo, Molèkangan, and Idilèrou appeared as cultivars with high dry matter content.

To compare farmers' and processors' technological perceptions that have been accessed to physicochemical composition of the sample, fresh cassava roots of G1 cultivars were analyzed for dry matters, fiber, starch, total sugars, and cyanide content and the results are presented in Table 4. The highest rate of dry matter (44.67%), starches (25.5%), fibers (1.02%), total sugars (41.13%), and cyanides (50.24 mg/kg) content was recorded for Molekangan while Odohoungbo showed, apart from total sugars, the lowest values for all the parameters considered. However, no significant difference (*P* > 5%) among the three most preferred cultivars was found as far as the starch and cyanide content. The dry matter content of Idilèrou (43.15%) was not significantly different to the one of Odohoungbo (42.34%), while Molèkangan showed significantly higher dry matter content. The results are similar to those (37.30% to 45.26%) reported by Fakir et al. (2012) in Bangladesh and higher than the values (28.8% to 41.1%) obtained, respectively, by [1, 15] on six cassava cultivars in Benin. As reported by [24], this difference might be due to variations in genotypes and in the growing conditions of the cultivars. High dry matter of cassava roots could contribute to the increase of the yield and the texture of derivative product [8]. Consequently, the higher dry matter of the cultivars probably explained their high aptitude for in-ground postmaturity storage, high yield, and good texture of ends products previously reported in this paper. This result was confirmed by farmers who noted that the higher the dry matter content of cassava cultivar, the better the yield of derived gari and tapioca. From this finding, it is shown that surveyed farmers have good perception on dry matter content of elite most preferred cassava cultivars.

The starches content of the three cultivars (Idilèrou, Odohoungbo, and Molèkangan) on wet weigh basis were 24.7%, 24.47%, and 25.5%, respectively. These values are greater than the values ranged from 15.04 to 21.91% obtained by [24] for five accessions of cassava. However, these results are comparable to the values of 24.90% to 25.05% obtained by the same authors for other cultivars.

The fibers content varied significantly (*P* ≤ 0.05) between the three cassava cultivars analyzed. The values obtained ranged from 0.80 to 1.02% and they were lower than the values of 1.60% reported by [5] and 1.66 to 4.27% reported by [24] for seven accessions of cassava in Bangladesh. However, Molèkangan cultivars fiber content (1.02%) had similar value as to the one (1.02%) obtained by [1] in Nigeria. Regarding these low fibers' content of Idilèrou, Odohoungbo, and Molèkangan cassava cultivars revealed by physicochemical analysis, it could be concluded that farmers and processors have good perception of fibers content of their cultivars.

The total sugars content on fresh weight basis is 40.20%, 39.46%, and 41.13% for Idilèrou, Odohoungbo, and Molèkangan cassava cultivars, respectively. These are in agreement with Balamurugan and Anbuselvi [5] who reported that cassava is a rich source of carbohydrates. The three cultivars were significantly different in terms of total sugars content.

The cyanide content of three cultivars analyzed ranging from 50.13 to 50.24 mg/kg are very low compared to the cyanides content values: 132 mg/kg 91.6 to 189 mg/kg, and 137.20 to 546.01 mg/kg, reported, respectively, by [1, 15] for six cassava cultivars in Benin and by [24] for seven accessions of cassava in Bangladesh. This observation may be due to the fact that the cyanide content of cassava is known to vary with environmental conditions [11, 12, 25] and with genetic factors [11, 25].

Although there is no general consensus on the safe levels of cyanide for both human and animal consumption [12], it is noted that a great danger of chronic poisoning might occur if roots with more than 150 mg HCN per kg is consumed. According to [12], when the peeled portion contains <50 mg HCN per kg of freshly grated cassava, the cassava is considered innocuous and can be taken as harmless to the consumer. A concentration between 50 mg and 80 mg/kg may be slightly poisonous; 80–100 mg/kg is toxic while concentrations above 100 mg/kg of grated cassava are dangerously poisonous [11, 25]. In regard to these guidelines, it can be observed that the cyanide content values of the three cassava cultivars analyzed were slightly higher than 50 ppm, the International Codex Standard upper limit in “sweet cassava.” According to [15], cultivar with low cyanide content also had the lowest sugar content and despite its low cyanide content was scored as the most bitter. This finding is in agreement with the observation of the results of physicochemical analysis especially for the cultivars Idilèrou, Odohoungbo, and Molèkangan and also revealed that farmers surveyed have good knowledge on cyanide content of cassava cultivars according to their perception of their bitter taste. Even though these three cultivars could be ranged among slightly poisonous cassava cultivars, it is generally known that cyanide could almost be removed by proper processing of cassava root [24]. The gari and tapioca thus produced was free of toxicity since cyanide content was not detected.

### 3.3. Sensory Properties of Gari and Tapioca Derived from the Three Selected Cultivars

Table 5 presented the sensory profiles of gari and tapioca from each of three selected cassava cultivars. There is no significant difference between cultivars Idilèrou and Odohoungbo in terms of taste of gari and taste of tapioca. The gari from cultivars Idilèrou and Odohoungbo appears sweet for the majority (73.67%) of the tasters while their tapioca is very sweet for 60% of panelists. Based on their higher sweetness scored by panelist, cultivars Idilèrou and odohoungbo were considered rich in soluble sugars while they contain lower amount of sugars and appeared as the bitterest. These results were in agreement with those of Hongbete et al. (2011) who reported that bitter cultivars had higher soluble sugar contents than sweet ones and bitter cultivars from Amazonia have also been found to give sweeter products. These observations corroborated the classification used by Bantè farmers.

For 80% and 56.67% of tasters, the cultivar Idilèrou and odohoungbo present “white” gari while cultivar Monlèkangan gari presents “dirty white” color for the majority (56.67%) of panelists. The tapioca from Odohoungbo was perceived as the whitest (90%) by tasters. The dirty white color of gari from Monlèkangan may be due to its high no soluble sugars content which tends to caramelize during frying step.

Among the products from the three cultivars, gari and tapioca from Odohoungbo recorded the major ratio for “very dry” by tasters (60% and 36.67%). According to the observation of [1], low fibers content of cassava could be correlated to good yield of gari. This latest affirmation could be correlated to the results of farmer's perception on technological properties of the cassava cultivars in the study area. The texture of gari and tapioca could be associated to the low fibers content of the cassava cultivars on the one hand. On the order hand, the lower the starches content of cassava cultivars the more important the water removing activity (due to the limitation of the ratio of gelatinization of starches) from the product during the frying step which leads to very dry (most drier) products.

## 4. Conclusion

This study allowed us to document the cassava cultivars diversity in the district of Bantè and to identify the elite and the most preferred cultivars. To select cassava cultivars to be produced or to buy in advance material for processing, farmers rely most on high productivity and good in-ground postmaturity storage aptitude. Therefore, these two criteria must be taken into consideration for cassava breeding program in Benin. The data of the physicochemical and sensory analysis of the three most preferred elite cultivars are well correlated with the farmers' perceptions on agronomic, culinary, and technological properties of cassava cultivars. Therefore, their knowledge should be capitalized by food technologists and breeders. For better development of the cassava value chain in Benin, there will be a need to identify duplicates and clarify synonymies using molecular markers; assess the proximate, mineral, and vitamins A and C compositions of all the elites cultivars; and evaluate the suitability of these cultivars for the production of the other major consumed food products such as high quality cassava flours for baking and complementary infant's flour.

## Figures and Tables

**Table 1:** Diversity, distribution, and extent of cassava cultivars in Bantè district.

Number	Cultivars	Cycle (month)	Village, distribution and extent
1	Abakokota	12	Kafégnigbé (− −)
2	Adobia	7–12	Akatakou (+−); Assaba (+−); Banon (+−); Djagbalo (+−); Lougba (+−); Okoutaossé (−+)
3	Agrik	12	Banon (− −); Djagbalo (+−); Lougba (−+); Okoutaossé (+−)
4	Akpalokofo	12	Assaba (+−); Banon (−+); Kafégnigbé (−+)
5	Aroubièlou	8–12	Assaba (−+)
6	Assoumon	6–8	Akatakou (+−)
7	Boconnon	12	Banon (− −)
8	Bamiwômô	7–10	Assaba (− −)
9	Banchiognibo	8–12	Akatakou (− −); Banon (− −); Djagbalo (+−); Kafégnigbé (+−); Okoutaossé (− −)
10	BEN	6–10	Akatakou (−+); Assaba (++)
11	Dangariya	12	Assaba (−+)
12	Doufè	12	Assaba (− −)
13	Êguèdoudou	12	Adja Pira (−+); Assaba (+−); Okoutaossé (− −)
14	Gbamilôya	6	Banon (− −); Lougba (− −)
15	Ghana	18	Lougba (− −); Assaba (− −)
16	Idilèrou	12–18	Akatakou (−+); Assaba (− −); Djagbalo (++)
17	Onichoutin	10–12	Assaba (+−); Akatakou (− −); Banon (− −); Djagbalo (−+); Lougba (+−)
18	Kalaba	12	Assaba (− −)
19	Katapila	12	Lougba (− −)
20	Kinkélédji	12	Akatakou (− −); Assaba (+−)
21	Konkonni	18	Banon (− −)
22	Koukpabiékpo	10–12	Assaba (− −)
23	Maboussa	10–12	Adja Pira (++); Akatakou (+−); Assaba (++); Banon (++); Djagbalo (+−); Lougba (++); Okoutaossé (++)
24	Malèbra	12	Assaba (− −)
25	Monlèkagan	12	Adja Pira (− −); Assaba (++)
26	Nontékponmi	6	Lougba (− −)
27	Obia	12–18	Kafégnigbé (−+)
28	Odohoungbo	12–18	Adja Pira (++); Akatakou (++); Assaba (++); Banon (++); Djagbalo (++); Kafégnigbé (++); Lougba (++); Okoutaossé (++)
29	Ogoubi	6–10	Adja Pira (+−); Akatakou (− −); Assaba (− −); Banon (−+); Lougba (+−)
30	Ôhôyi	12	Kafégnigbé (−+)
31	Ôkôyawo	12	Adja Pira (++); Akatakou (++); Assaba (++); Banon (++); Djagbalo (++); Kafégnigbé (++); Lougba (+−); Okoutaossé (++)
32	Ôlôbêkpê	9–12	Adja Pira (+−); Akatakou (+−); Assaba (+−); Banon (+−); Djagbalo (−+); Kafégnigbé (+−); Lougba (+−); Okoutaossé (− −)
33	RB	6–12	Akatakou (− −); Assaba (++); Kafégnigbé (++)
34	Tataboé	9	Banon (− −)
35	Tatawi	6	Akatakou (− −); Assaba (++); Djagbalo (− −)
36	Tchabaou	7–10	Kafègnigbé (− −)
37	Tchamba	12	Assaba (− −)
38	Tiôka	12	Assaba (− −); Banon (+−); Lougba (+−); Okoutaossé (+−)
39	TMS	8–10	Akatakou (++); Assaba (++); Djagbalo (+−)
40	Yaouïdalè	12–18	Lougba (− −); Okoutaossé (− −)

**Table 2:** Agronomic, culinary, and technology characteristic of elite cultivars.

Cultivars	Prt	MCy	GPS	Tas	Fco	YGa	QGa	Sco	QTa	QDo
BEN	Hi	Lm	Ba	Su	Lo	Hi	Go	Hi	Go	Go
Idilerou	Hi	Vl	Go	Bi	Lo	Hi	Go	Hi	Go	Go
Maboussa	Av	Lm	Go	Su	Lo	Hi	Go	Hi	Go	Go
Monlèkangan	Hi	Vl	Go	Bi	Lo	Hi	Go	Hi	Go	Go
Odohoungbo	Hi	Vl	Go	Bi	Lo	Hi	Go	Hi	Go	Go
Ôkôtiyawo	Hi	Em	Ba	Su	Lo	Hi	Go	Hi	Go	Go
RB	Hi	Em	Ba	Su	Lo	Hi	Go	Hi	Go	Go
Tatawili	Av	Em	Go	Su	Lo	Hi	Go	Lo	Go	Go
TMS	Hi	Lm	Ba	Su	Lo	Hi	Go	Hi	Go	Go

Prt: productivity; MCy: maturity cycle; GPS: in-ground postmaturity storage; Tas: taste; Fco: fiber content; YGa: yield of gari; QGa: quality of gari; Sco: starch content; QTa: quality of tapioca; and QDo: quality of dough.

Hi: High; Av: average; Lm: late maturity; Vl: very late maturity; Em: early maturity; Ba: bad; Su: sugared; Bi: bitter; Go: good; Lo: low.

**Table 3:** Farmers and processors perceptions on physicochemical properties of the roots of G1 cassava cultivars.

Evaluation parameters	Cultivars	Farmers' and processors' perceptions	
		Low (%)	High (%)
Fibers content	Idilèrou	92.68	7.32
	Odohoungbo	100	0
	Monlèkangan	95	5

Starches content	Idilèrou	29.27	70.73
	Odohoungbo	0	100
	Monlèkangan	5	95

Dry matter content	Idilèrou	17.07	82.93
	Odohoungbo	0	100
	Monlèkangan	0	100

Cyanide content	Idilèrou	0	100
	Odohoungbo	0	100
	Monlèkangan	0	100

**Table 4:** Physicochemical composition of the three most preferred cassava elite cultivars.

Chemical composition	Idilèrou	Odohoungbo	Molèkangan
Starches (WW) (%)	24.70 ± 0.10^a^	24.47 ± 0.49^a^	25.5 ± 0.02^a^
Dry matters (%)	43.15 ± 0.26^a^	42.34 ± 0.42^a^	44.67 ± 0.21^b^
Fibers (%)	0.90 ± 0.010^a^	0.80 ± 0.02^b^	1.02 ± 0.010^c^
Cyanide (mg/kg)	50.19 ± 0.04^a^	50.13 ± 0.03^a^	50.24 ± 0.04^a^
Total sugars (WW) (%)	40.20 ± 0.10^b^	39.46 ± 0.20^a^	41.13 ± 0.03^c^

The value with the same superscript letter in the row are not significantly different from each other at probability *P* = 0.05.

**Table 5:** Sensory profiles of gari and tapioca from the three most preferred cassava elite cultivars.

Products	Cultivars	Taste		Color		Texture	
		Sw%	VSw%	Wh%	BWh%	Dr%	VDr%
Gari	Idilèrou	76.67	23.33	80.00	20.00	60.00	40.00
	Odohoungbo	76.67	23.33	56.67	43.33	40.00	60.00
	Monlèkangan	83.33	16.67	43.33	56.67	73.33	26.67

Tapioca	Idilèrou	40.00	60.00	73. 33	26.67	80	20
	Odohoungbo	40.00	60.00	90.00	10.00	63.33	36.67
	Monlèkangan	63.33	36.67	76.67	23.33	76.67	23.33

Sw: sweet; VSw: very sweet; Wh: white; BWh: brownish white; Dr: dry; VDr: very dry.
